# Association of frailty with the incidence risk of cardiovascular disease and type 2 diabetes mellitus in long-term cancer survivors: a prospective cohort study

**DOI:** 10.1186/s12916-023-02774-1

**Published:** 2023-02-24

**Authors:** Xingqi Cao, Zhenqing Yang, Xueqin Li, Chen Chen, Emiel O. Hoogendijk, Jingyun Zhang, Nengliang Aaron Yao, Lina Ma, Yawei Zhang, Yong Zhu, Xuehong Zhang, Yuxian Du, Xiaofeng Wang, Xifeng Wu, Thomas M. Gill, Zuyun Liu

**Affiliations:** 1 Center for Clinical Big Data and Analytics of the Second Affiliated Hospital and Department of Big Data in Health Science School of Public Health, The Key Laboratory of Intelligent Preventive Medicine of Zhejiang Province, Zhejiang University School of Medicine, 866 Yuhangtang Rd, Zhejiang, 310058 Hangzhou China; 2 China CDC Key Laboratory of Environment and Population Health, National Institute of Environmental Health, Chinese Center for Disease Control and Prevention, Beijing, 100000 China; 3 Department of Epidemiology & Data Science, Amsterdam Public Health research Institute, Amsterdam UMC – location VU University Medical Center, P.O. Box 7057, 1007MB, Amsterdam, the Netherlands; 4 Home Centered Care Institute, Schaumburg, IL USA; 5 Center For Health Management and Policy, School of Public Health, Cheeloo College of Medicine, Shandong University, Jinan, 250012 China; 6 Section of Geriatrics, University of Virginia, Charlottesville, VA USA; 7 Department of Geriatrics, Xuanwu Hospital Capital Medical University, National Clinical Research Center for Geriatric Diseases, Beijing, 100053 China; 8 Beijing Geriatric Healthcare Center, Xuanwu Hospital Capital Medical University, Beijing, 100053 China; 9 National Cancer Center/National Clinical Research Center for Cancer/Cancer Hospital, Chinese Academy of Medical Sciences and Peking Union Medical College, Beijing, 100021 China; 10 Department of Environmental Health Sciences, Yale School of Public Health, Yale University, New Haven, CT 06510 USA; 11 Channing Division of Network Medicine, Department of Medicine, Brigham and Women’s Hospital and Harvard Medical School, Boston, MA 02115 USA; 12 Bayer Healthcare Pharmaceuticals U.S. LLC, Whippany, NJ 07981 USA; 13 State Key Laboratory of Genetic Engineering, Collaborative Innovation Center for Genetics and Development, School of Life Sciences and Human Phenome Institute, Fudan University, Shanghai, 200433 China; 14 National Clinical Research Center for Ageing and Medicine, Huashan Hospital, Fudan University, Shanghai, 200040 China; 15 Department of Internal Medicine, Yale School of Medicine, New Haven, CT 06511 USA

**Keywords:** Cancer survivors, Frailty, Aging, Cardiovascular disease, Type 2 diabetes mellitus

## Abstract

**Background:**

Comorbidities among cancer survivors remain a serious healthcare burden and require appropriate management. Using two widely used frailty indicators, this study aimed to evaluate whether frailty was associated with the incidence risk of cardiovascular disease (CVD) and type 2 diabetes mellitus (T2DM) among long-term cancer survivors.

**Methods:**

We included 13,388 long-term cancer survivors (diagnosed with cancer over 5 years before enrolment) free of CVD and 6101 long-term cancer survivors free of T2DM, at the time of recruitment (aged 40–69 years), from the UK Biobank. Frailty was assessed by the frailty phenotype (FP_Frailty, range: 0–5) and the frailty index (FI_Frailty, range: 0–1) at baseline. The incident CVD and T2DM were ascertained through linked hospital data and primary care data, respectively. The associations were examined using Cox proportional hazards regression models.

**Results:**

Compared with non-frail participants, those with pre-frailty (FP_Frailty [met 1–2 of the components]: hazard ratio [HR]=1.18, 95% confidence interval [CI]: 1.05, 1.32; FI_Frailty [0.10< FI ≤0.21]: HR=1.51, 95% CI: 1.32, 1.74) and frailty (FP_Frailty [met ≥3 of the components]: HR=2.12, 95% CI: 1.73, 2.60; FI_Frailty [FI >0.21]: HR=2.19, 95% CI: 1.85, 2.59) had a significantly higher risk of CVD in the multivariable-adjusted model. A similar association of FI_Frailty with the risk of incident T2DM was observed. We failed to find such an association for FP_Frailty. Notably, the very early stage of frailty (1 for FP_Frailty and 0.1-0.2 for FI_Frailty) was also positively associated with the risk of CVD and T2DM (FI_Frailty only). A series of sensitivity analyses confirmed the robustness of the findings.

**Conclusions:**

Frailty, even in the very early stage, was positively associated with the incidence risk of CVD and T2DM among long-term cancer survivors, although discrepancies existed between frailty indicators. While the validation of these findings is required, they suggest that routine monitoring, prevention, and interventive programs of frailty among cancer survivors may help to prevent late comorbidities and, eventually, improve their quality of life. Especially, interventions are recommended to target those at an early stage of frailty when healthcare resources are limited.

**Supplementary Information:**

The online version contains supplementary material available at 10.1186/s12916-023-02774-1.

## Background

About half of the UK population born after 1960 are expected to be diagnosed with cancer during their lifetime [1]. With the advance of treatment techniques and effective pharmaceutical agents, the 10-year survival rate of cancer patients has reached 50% over the past several decades in the UK [1, 2]. In comparison with the non-cancer population, cancer survivors are at a 13–70% and 17–158% higher risk of developing cardiovascular disease (CVD) [3] and type 2 diabetes mellitus (T2DM) [4], respectively. Moreover, dying of CVD is common among survivors of site-specific cancer (e.g., breast cancer [5] and prostate cancer [6]), and the corresponding probability is even higher than that of dying from cancer per se [7]. Given the adverse effects of CVD and T2DM on survival and quality of life among cancer survivors [8], finding prognostic factors is crucial to help identify the vulnerable subgroup and then implement appropriate disease management and therapeutic interventions in clinical practice [9, 10].

Previous studies on the prognostic factors of cancer survivors have mainly focused on biological biomarkers [11, 12] and lifestyle [13, 14]. Increasing attention has recently been drawn to the accelerated fundamental aging process, which is one of the most significant characteristics of cancer survivors [15, 16]. In other words, these patients experience an accelerated decline in the trajectories of physical, cognitive, and psychosocial morbidities [17], which are largely captured in the concept of frailty. Frailty is characterized by an increased vulnerability to stressor events caused by cumulative diminished reserve and dysregulation in multiple physiological systems [18] and has been widely used as a valid indicator of the aging process in geriatrics and gerontology [19, 20]. There are two principal frailty indicators: the frailty phenotype proposed by Fried et al. (FP_Frailty) [18] and the frailty index (FI) proposed by Mitnitski et al. (FI_Frailty) [21]. Frailty is usually accompanied by the dysregulation of metabolic and endocrine systems [20, 22], which may further contribute to CVD and T2DM in the general population [23, 24]. About 42% (range: 6–86%) of older cancer survivors exhibit frailty [25], and this proportion is nearly four times higher than that in the community-based older population [26]. Although frailty has been used to predict poor outcomes including mortality among cancer survivors [17, 27], it remains unclear whether it increases the risk of CVD and T2DM in this group. If this is the case, screening cancer survivors for frailty, particularly those at an early stage of frailty (which is potentially reversible) [18], may provide opportunities to delay or prevent the occurrence of late comorbidities, and thus improve their quality of life.

**Fig. 1:**
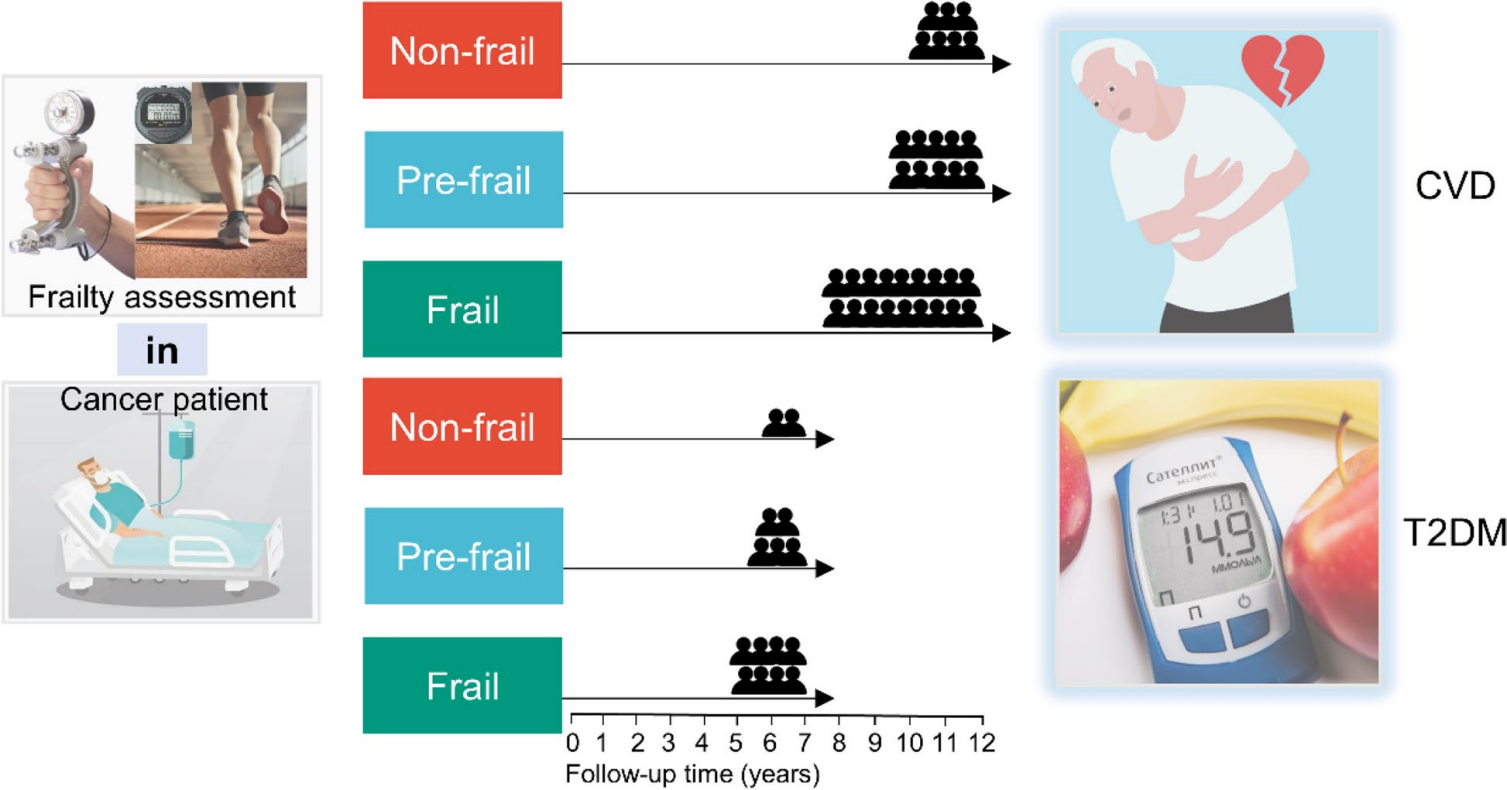
The influence of frailty on the incidence risk of CVD and T2DM during follow-up among long-term cancer survivors from the UK Biobank. CVD, cardiovascular disease, T2DM, type 2 diabetes mellitus. The median follow-up years for CVD and T2DM were 12 and 7 years, respectively

In this study, we included 13,388 long-term cancer survivors (diagnosed with cancer five or more years earlier) free of CVD and 6101 long-term cancer survivors free of T2DM at the time of recruitment, based on data from the UK Biobank (UKB). Given that FP_Frailty and FI_Frailty are complementary [28], the present study aimed to evaluate whether both frailty indicators were significantly associated with incident CVD and T2DM in the large samples of long-term cancer survivors (Fig. 1).

## Methods

### Study participants

The UKB is a prospective cohort study that recruited approximately 500,000 participants aged 40–69 years in the UK between 2006 and 2010 [29]. Those living near the assessment centers across most of the UK were recruited within 6–12 months through an invitation from population-based registers (such as those held by the National Health Service). The UKB database was granted approval by the North West Multi-center Research Ethics Committee. Written informed consent was obtained from all participants in UKB. In this study, the diagnosis information of cancer (excluding non-melanoma skin cancer) was provided by the Medical Research Information Service of the National Health Service (NHS) Information Centre (residents in England and Wales) and the Information Services Division of NHS Scotland (residents in Scotland) (see details at https://biobank.ndph.ox.ac.uk/showcase/showcase/docs/CancerLinkage.pdf) using the International Statistical Classification of Diseases and Related Health Problems 9^th^ (ICD-9) (140-208, except 173 [i.e., non-melanoma skin cancer]) and 10^th^ edition (ICD-10) (C00-C96, except C44 [i.e., non-melanoma skin cancer]). At baseline, there were 26,543 cancer survivors, of which 15,289 had been diagnosed with cancer more than 5 years earlier, i.e., they were regarded as long-term cancer survivors. Participants were excluded if they had missing data on frailty measures (i.e., five FP_Frailty components or ≥10 FI_Frailty items; *N*=45) or covariates (*N*=791). Additionally, we excluded those with prevalent CVD at the time of recruitment (*N*=1065) for analysis of incident CVD and excluded those with prevalent diabetes at the time of recruitment (*N*=947) and without primary care data (*N*=7405) for analysis of incident T2DM, respectively. The final analytic samples included 13,388 long-term cancer survivors for analysis of incident CVD and 6101 long-term cancer survivors for analysis of incident T2DM (Additional file 1: Fig. S1).

### Outcomes

The primary disease outcomes of this study were CVD and T2DM. The prevalent CVD was assessed by linked records and self-reported history. The prevalent diabetes was assessed by self-reported data from touchscreen questionnaire and nurse verbal interview [30], as well as linked records. The incident CVD was ascertained through linked hospital data until Aug 29, 2021. The incident T2DM was ascertained through primary care data until 2016/2017 (England: May 31, 2016/2017; Scotland: Mar 31, 2017; Wales: Aug 31, 2017). We used ICD-9 (CVD: 410, 411, 412, 413, 414, 429.79, 430, 431, 432, 433, 434, 435, 436, 437, and 438; T2DM: 250.00, 250.10, 250.20, and 250.90) and ICD-10 (CVD: I20, I21, I22, I23, I24.1, I25, I46, I60, I61, I63, and I64; T2DM: E11) to define CVD [31] and T2DM [30, 32]. The time-to-event for participants was calculated from the date of recruitment (between 2006 and 2010) to the date of the first diagnosis of CVD or T2DM, date of death, date of loss to follow-up, or end of follow-up, whichever came first.

### Predictors: frailty

Both FP_Frailty and FI_Frailty were measured at baseline. FP_Frailty measured physical frailty and was assessed using five self-reported or objectively measured components, including low energy expenditure, exhaustion, slow gait speed, weakness, and unintentional weight loss (see details in Additional file 2: Supplementary Methods [18, 33]). Based on the results, participants were classified as non-frail (if they met none of the criteria), pre-frail (if they met 1–2 of the criteria), and frail (if they met ≥3 of the criteria), if they did not have missing data on all five components.

FI_Frailty reflected the cumulative health deficits and was assessed using 49 self-reported items, including deficits in the sensory, cranial, mental wellbeing, infirmity, cardiometabolic, respiratory, musculoskeletal, immunological, cancer, pain, and gastrointestinal domains (see details in Additional file 3: Table S1) [34]. The ratio of the number of deficits to total possible deficits was calculated as a FI score (ranged from 0-1) for each participant [35] with missing data on less than 10 FI_Frailty items. Participants were classified as non-frail (FI score ≤0.10), pre-frail (0.10< FI score ≤0.21), and frail (FI score >0.21) [36].

In the primary analysis, we used the three-category variables (i.e., non-frail, pre-frail, and frail) for both FP_Frailty and FI_Frailty. Additionally, for the secondary analysis, we divided the FP_Frailty score into five categories: 0, 1, 2, 3, and ≥4, and the FI_Frailty score into four categories: [0, 0.1], [0.1, 0.2], [0.2, 0.3], and [0.3, ~]. Participants who met 1 of the components of FP_Frailty, or had an FI score between 0.1 and 0.2 were pre-frail, which could be interpreted as an early stage of frailty.

### Covariates

Information on age, sex, ethnicity, educational attainment, occupational status, alcohol consumption, smoking status, regular exercise, and family histories of CVD and diabetes was collected through the questionnaire at baseline. Ethnicity included White, Mixed, Asian, Black, Chinese, and other backgrounds. Educational attainment was classified as high (college or university degree), intermediate (A/AS levels or equivalent, O levels/General Certificate of Secondary Education levels or equivalent), and low (none of the above). The occupational status was classified as working, retired, and other (including unemployed, student, volunteer, and so on). Alcohol consumption was categorized as never or occasional, one to three times per month, one to four times per week, and daily. The smoking status was categorized as non-smoker, ever-smoker, and current smoker. Regular exercise was classified as yes if participants undertook 75 min of vigorous activity or 150 min of moderate activity or an equivalent combination thereof per week [37]. The family histories of CVD and diabetes were classified as yes or no. Townsend deprivation index (TDI) was assigned based on participants’ postcodes. The TDI measured socioeconomic status levels, with a lower score indicating a higher level of socioeconomic status [38]. The measured body mass index (BMI) in kg/m^2^ was derived at baseline.

### Statistical analyses

We presented the baseline characteristics of the analytic samples in total and by incident CVD and T2DM, with numbers and percentages for categorical variables, and medians and inter-quartile ranges (IQRs) for continuous variables. The characteristics of incident CVD or T2DM were compared using *χ*^2^ and Kruskal-Wallis tests for categorical and continuous variables, respectively.

The differences in cumulative CVD and T2DM hazards among categories by FP_Frailty and FI_Frailty were compared through Kaplan-Meier curves. To evaluate the associations of frailty with incident CVD and T2DM among cancer survivors, we used Cox proportional hazards regression models. The hazard ratios (HRs) and corresponding 95% confidence intervals (CIs) were calculated from two models. Model 1 adjusted for age and sex, while model 2 additionally adjusted for ethnicity, educational attainment, occupational status, TDI, alcohol consumption, smoking status, regular exercise, BMI, and family history of CVD (or diabetes). Non-frail participants were set as the reference group in the primary analysis, and the category of the lowest score of FP_Frailty (and FI_Frailty) was set as the reference in the secondary analysis. Schoenfeld residuals test was used to verify the proportional hazard assumption, and no significant violation was found.

In the sensitivity analyses, we first repeated the primary analysis by (1) including all cancer survivors (i.e., adding those diagnosed less than five years from enrolment); (2) excluding survivors who were lost to follow-up, deceased, or diagnosed with incident CVD, within the first 2 years from baseline, to minimize the impact of reverse causality; (3) setting the end of follow-up at the end of 2019 to reduce the influence of COVID-19 (for incident CVD only); (4) defining incident T2DM using primary and secondary care (i.e., linked hospital data) data simultaneously; (5) assessing the competing risk from all-cause death using the Fine-Gray model with consideration of the high mortality among cancer survivors [39]; (6) and further adjusting for important risk factors for CVD and T2DM, including blood pressure, glucose, and lipids levels. Second, (1) to account for the influence of seven CVD-, diabetes- or cancer-related items in the FI, we excluded these items to construct a modified FI; (2) we used different cut-off values of the FI score to define frailty, i.e., non-frail: FI score ≤0.10, pre-frail: 0.10< FI score <0.25, and frail: FI score ≥0.25 [40], and we then repeated the primary analysis for FI_Frailty. Third, the component of low energy expenditure in FP_Frailty was similar to a covariate (i.e., regular exercise). Therefore, we removed regular exercise from model 2 and repeated the primary analysis of FP_Frailty. Fourth, to minimize the influence of the missing data on our estimates, we repeated the primary analysis using a sample with complete data on all frailty components (or items). Finally, HRs were also calculated for individual components included in FP_Frailty using the same set of covariates as in model 2.

In this study, *P* values less than 0.05 were considered statistically significant. All statistical analyses were conducted in R version 3.6.3 (2020-02-29) and SAS version 9.4 (SAS Institute, Cary, NC).

## Results

### Baseline characteristics

The median ages of the 13,388 and 6101 recruited participants were 61.9 (IQR: 56.4, 65.7) years and 62.1 (IQR: 56.8, 65.8) years, respectively. The majority were female (72.0% and 71.7%) and White (97.6% and 98.4%). About 40% and 8% of participants were survivors of breast and colorectal cancer, respectively. During the follow-up, 10.3% (1380/13,388) of the participants developed CVD, and 3.1% (187/6,101) of participants developed T2DM. Compared with participants who did not develop these two conditions during follow-up, those with CVD or T2DM were more likely to be older and male. The detailed baseline characteristics of the study participants are shown in Table 1.

**Table 1:** Baseline characteristics of the study participants in total and by incident CVD and T2DM

Variable	Analytic sample 1				Analytic sample 2			
	Total (***N***=13,388)	No CVD (***N***=12,008)	CVD (***N***=1380)	***P*** value	Total (***N***=6101)	No T2DM (***N***=5914)	T2DM (***N***=187)	***P*** value
Cancer type^a^								
Breast cancer	5500 (41.1)	5092 (42.4)	408 (29.6)	<0.001	2488 (40.8)	2418 (40.9)	70 (37.4)	0.365
Colorectal cancer	1064 (8.0)	938 (7.8)	126 (9.1)	0.086	524 (8.6)	511 (8.6)	13 (7.0)	0.507
Prostate cancer	958 (7.2)	793 (6.6)	165 (12.0)	<0.001	427 (7.0)	406 (6.9)	21 (11.2)	0.028
Kidney cancer	208 (1.6)	172 (1.4)	36 (2.6)	0.001	115 (1.9)	107 (1.8)	8 (4.3)	0.024
Other	5773 (43.1)	5118 (42.6)	655 (47.5)	<0.001	2598 (42.6)	2522 (42.6)	76 (40.6)	0.600
Age, years	61.9 (56.4, 65.7)	61.7 (56.0, 65.5)	63.6 (59.8, 66.9)	<0.001	62.1 (56.8, 65.8)	62.0 (56.7, 65.8)	63.7 (60.0, 67.2)	<0.001
Sex				<0.001				<0.001
Female	9641 (72.0)	8874 (73.9)	767 (55.6)		4376 (71.7)	4264 (72.1)	112 (59.9)	
Male	3747 (28.0)	3134 (26.1)	613 (44.4)		1725 (28.3)	1650 (27.9)	75 (40.1)	
Ethnicity				0.142				0.002
White	13,072 (97.6)	11,733 (97.7)	1339 (97.0)		6005 (98.4)	5827 (98.5)	178 (95.2)	
Mixed background	46 (0.3)	41 (0.3)	5 (0.4)		14 (0.2)	12 (0.2)	2 (1.1)	
Asian	91 (0.7)	74 (0.6)	17 (1.2)		31 (0.5)	27 (0.5)	4 (2.1)	
Black	92 (0.7)	84 (0.7)	8 (0.6)		25 (0.4)	24 (0.4)	1 (0.5)	
Chinese	23 (0.2)	21 (0.2)	2 (0.1)		7 (0.1)	6 (0.1)	1 (0.5)	
Other backgrounds	64 (0.5)	55 (0.5)	9 (0.7)		19 (0.3)	18 (0.3)	1 (0.5)	
Educational attainment				<0.001				0.007
High	4129 (30.8)	3782 (31.5)	347 (25.1)		1855 (30.4)	1812 (30.6)	43 (23.0)	
Intermediate	4378 (32.7)	3972 (33.1)	406 (29.4)		1926 (31.6)	1873 (31.7)	53 (28.3)	
Low	4881 (36.5)	4254 (35.4)	627 (45.4)		2320 (38.0)	2229 (37.7)	91 (48.7)	
Occupational status				<0.001				0.009
Working	5618 (42.0)	5175 (43.1)	443 (32.1)		2480 (40.6)	2424 (41.0)	56 (29.9)	
Retired	6751 (50.4)	5930 (49.4)	821 (59.5)		3172 (52.0)	3059 (51.7)	113 (60.4)	
Other^b^	1019 (7.6)	903 (7.5)	116 (8.4)		449 (7.4)	431 (7.3)	18 (9.6)	
Townsend deprivation index	-2.4 (-3.7, 0.0)	-2.4 (-3.7, -0.0)	-2.2 (-3.5, 0.6)	0.001	-2.4 (-3.8, 0.0)	-2.4 (-3.8, 0.0)	-2.4 (-3.6, 0.4)	0.493
Alcohol consumption				0.002				0.083
Never or occasional	2791 (20.8)	2459 (20.5)	332 (24.1)		1253 (20.5)	1202 (20.3)	51 (27.3)	
1–3 times/month	1464 (10.9)	1299 (10.8)	165 (12.0)		685 (11.2)	661 (11.2)	24 (12.8)	
1–4 times/week	6369 (47.6)	5771 (48.1)	598 (43.3)		2889 (47.4)	2811 (47.5)	78 (41.7)	
Daily	2764 (20.6)	2479 (20.6)	285 (20.7)		1274 (20.9)	1240 (21.0)	34 (18.2)	
Smoking status				<0.001				0.711
Non-smoker	7112 (53.1)	6510 (54.2)	602 (43.6)		3209 (52.6)	3116 (52.7)	93 (49.7)	
Ever-smoker	5044 (37.7)	4431 (36.9)	613 (44.4)		2332 (38.2)	2257 (38.2)	75 (40.1)	
Current smoker	1232 (9.2)	1067 (8.9)	165 (12.0)		560 (9.2)	541 (9.1)	19 (10.2)	
Regular exercise, yes	7111 (53.1)	6443 (53.7)	668 (48.4)	<0.001	3286 (53.9)	3213 (54.3)	73 (39.0)	<0.001
BMI, kg/m^2^	26.4 (23.8, 29.6)	26.3 (23.8, 29.5)	27.4 (24.6, 30.8)	<0.001	26.4 (23.8, 29.4)	26.3 (23.8, 29.3)	30.3 (27.7, 33.3)	<0.001
Family history of CVD, yes	8016 (59.9)	7127 (59.4)	889 (64.4)	<0.001	—	—	—	—
Family history of diabetes, yes	—	—	—	—	1183 (19.4)	1119 (18.9)	64 (34.2)	<0.001
FP_Frailty				<0.001				<0.001
Non-frail	6598 (49.3)	6016 (50.1)	582 (42.2)		3039 (49.8)	2977 (50.3)	62 (33.2)	
Pre-frail	6114 (45.7)	5460 (45.5)	654 (47.4)		2755 (45.2)	2647 (44.8)	108 (57.8)	
Frail	676 (5.0)	532 (4.4)	144 (10.4)		307 (5.0)	290 (4.9)	17 (9.1)	
FI_Frailty				<0.001				<0.001
Non-frail	4247 (31.7)	3965 (33.0)	282 (20.4)		1894 (31.0)	1872 (31.7)	22 (11.8)	
Pre-frail	7077 (52.9)	6324 (52.7)	753 (54.6)		3249 (53.3)	3140 (53.1)	109 (58.3)	
Frail	2064 (15.4)	1719 (14.3)	345 (25.0)		958 (15.7)	902 (15.3)	56 (29.9)	

The data are expressed as numbers and percentages for categorical variables and medians and inter-quartile range (IQR) for continuous variables. The *P* values were generated using *χ*^2^ and Kruskal-Wallis test for categorical and continuous variables, respectively

*CVD*, cardiovascular disease; *T2DM*, type 2 diabetes mellitus; *BMI*, body mass index; *FP_Frailty*, frailty defined by the frailty phenotype; *FI_Frailty*, frailty defined by the frailty index

^a^Percentages did not sum to 100 because some participants had more than one cancer diagnosis

^b^Other includes unemployed, student, volunteer, and so on

### The associations of frailty with incident CVD and T2DM

**Fig. 2:**
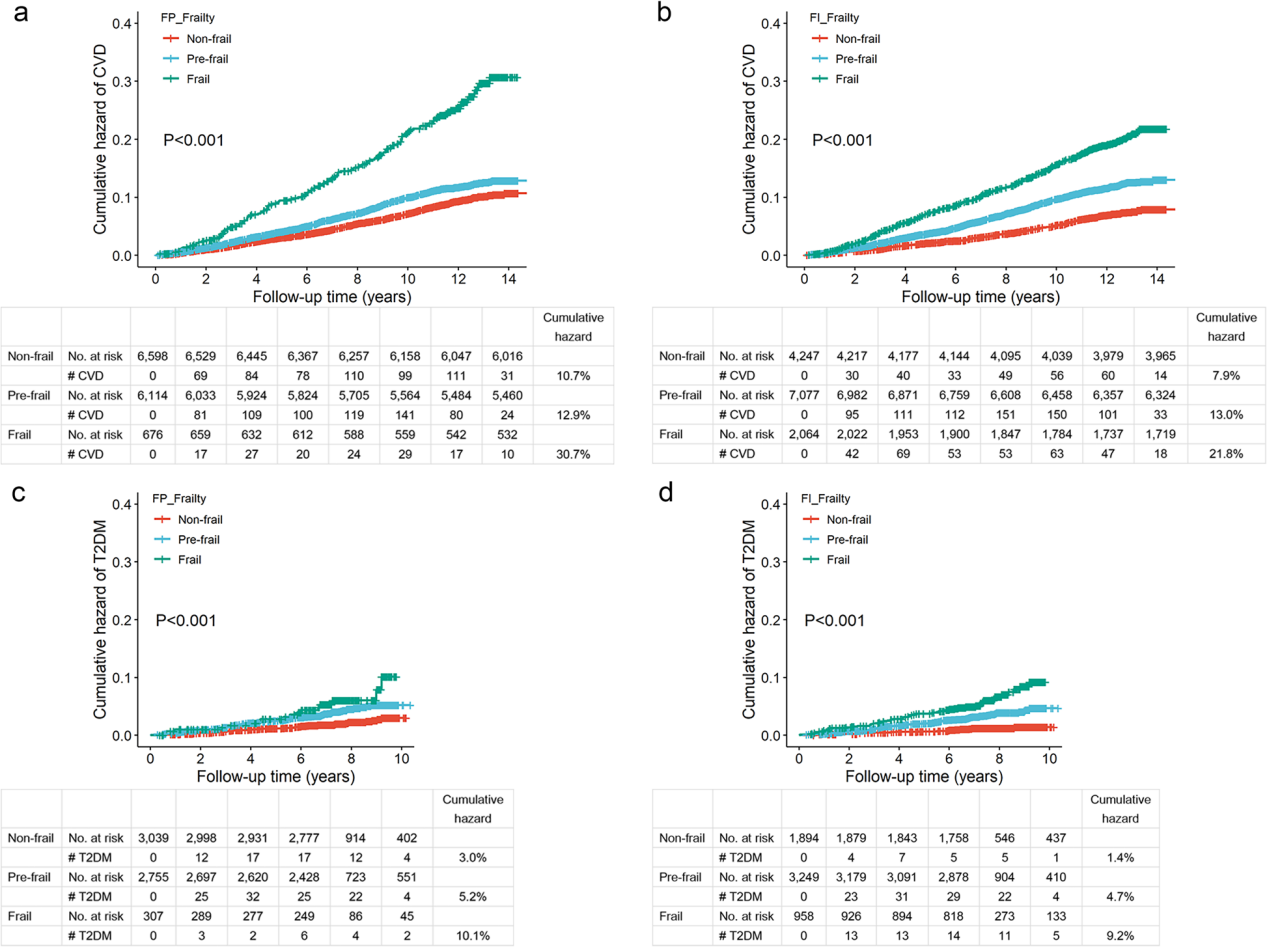
Kaplan-Meier curves of cumulative hazards of CVD and T2DM by three categories of frailty among long-term cancer survivors. CVD, cardiovascular disease; T2DM, type 2 diabetes mellitus; FP_Frailty, frailty defined by the frailty phenotype; FI_Frailty, frailty defined by the frailty index. **a** Kaplan-Meier curve of cumulative hazard of CVD for the study groups defined by the frailty phenotype. **b** Kaplan-Meier curve of cumulative hazard of CVD for the study groups defined by the frailty index. **c** Kaplan-Meier curve of cumulative hazard of T2DM for the study groups defined by the frailty phenotype. **d** Kaplan-Meier curve of cumulative hazard of T2DM for the study groups defined by the frailty index. The *y*-axis indicates the cumulative hazard of CVD or T2DM, and the *x*-axis indicates follow-up time in years

The Kaplan-Meier curves demonstrated the higher hazards for incident CVD and T2DM in participants with pre-frailty and frailty (Fig. 2). For instance, the cumulative hazard of CVD over a median of 12-year follow-up was the lowest in the category of non-frail (7.9–10.7%) and the highest in the category of frail (21.8–30.7%). Table 2 presents the associations of frailty with incident CVD and T2DM based on the primary analysis. Compared with non-frail participants, those with pre-frailty (FP_Frailty: HR=1.18, 95% CI: 1.05, 1.32; FI_Frailty: HR=1.51, 95% CI: 1.32, 1.74) and frailty (FP_Frailty: HR=2.12, 95% CI: 1.73, 2.60; FI_Frailty: HR=2.19, 95% CI: 1.85, 2.59) had a significantly higher risk of CVD in the multivariable-adjusted model. A similar association of FI_Frailty with the risk of incident T2DM was observed. We failed to find such an association for FP_Frailty.

**Table 2:** Primary analysis showing the associations of frailty (three-categorical) with incident CVD and T2DM among long-term cancer survivors.

	No. of events/No. of participants	Events/1000 person-years	Model 1	Model 2
			HR (95% CI)	HR (95% CI)
**CVD**				
**FP_Frailty**				
Non-frail	582/6598	7.7	Ref.	Ref.
Pre-frail	654/6114	9.6	1.31 (1.17, 1.46)	1.18 (1.05, 1.32)
Frail	144/676	21.3	2.97 (2.47, 3.57)	2.12 (1.73, 2.60)
*P* for trend	—	—	<0.001	<0.001
*P* for per 1-point increase	—	—	<0.001	<0.001
**FI_Frailty**				
Non-frail	282/4247	5.7	Ref.	Ref.
Pre-frail	753/7077	9.5	1.68 (1.46, 1.92)	1.51 (1.32, 1.74)
Frail	345/2064	15.7	2.83 (2.42, 3.32)	2.19 (1.85, 2.59)
*P* for trend	—	—	<0.001	<0.001
*P* for per 0.1-point increase	—	—	<0.001	<0.001
**T2DM**				
**FP_Frailty**				
Non-frail	62/3039	2.8	Ref.	Ref.
Pre-frail	108/2755	5.4	2.05 (1.50, 2.80)	1.46 (1.04, 2.05)
Frail	17/307	8.0	2.96 (1.73, 5.06)	1.29 (0.69, 2.39)
*P* for trend	—	—	<0.001	0.084
*P* for per 1-point increase	—	—	<0.001	0.083
**FI_Frailty**				
Non-frail	22/1894	1.6	Ref.	Ref.
Pre-frail	109/3249	4.6	2.89 (1.82, 4.59)	2.40 (1.51, 3.82)
Frail	56/958	8.2	5.18 (3.15, 8.53)	3.09 (1.81, 5.26)
*P* for trend	—	—	<0.001	<0.001
*P* for per 0.1-point increase	—	—	<0.001	<0.001

Linear trends were tested using FP_Frailty and FI_Frailty (three-categorical) as continuous variables

Model 1 was adjusted for age and sex

Model 2 was further adjusted for ethnicity, educational attainment, occupational status, Townsend deprivation index, alcohol consumption, smoking status, regular exercise, body mass index, and family history of CVD (or diabetes)

*CVD*, cardiovascular disease; *T2DM*, type 2 diabetes mellitus; *HR*, hazard ratio; *CI*, confidence interval; *FP_Frailty*, frailty defined by the frailty phenotype; *FI_Frailty*, frailty defined by the frailty index

**Fig. 3:**
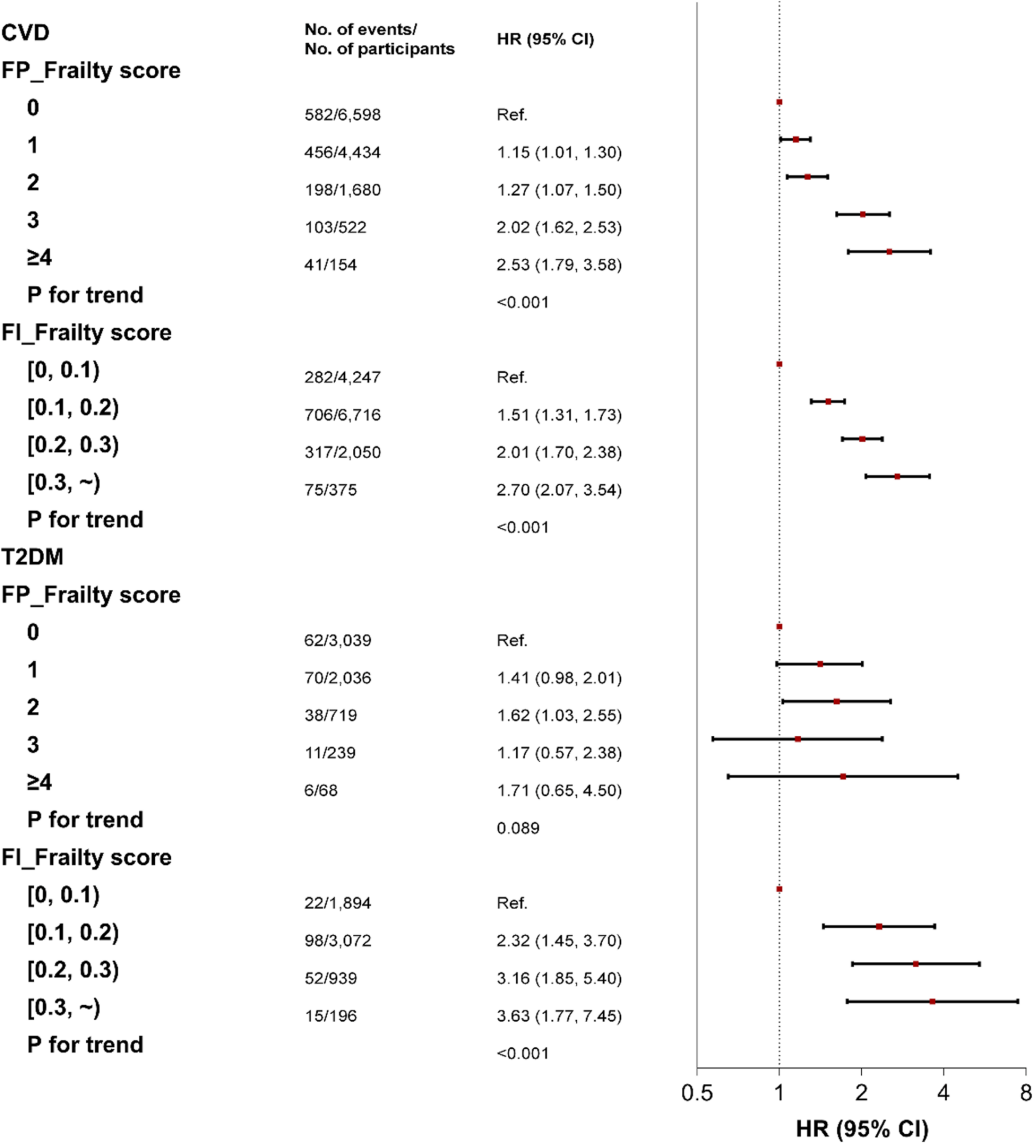
Secondary analysis showing the associations of frailty (score) with incident CVD and T2DM among long-term cancer survivors. CVD, cardiovascular disease; T2DM, type 2 diabetes mellitus; HR, hazard ratio; CI, confidence interval; FP_Frailty, frailty defined by the frailty phenotype; FI_Frailty, frailty defined by the frailty index. The *x*-axis was log_2_ scaled. The HRs were adjusted for age, sex, ethnicity, educational attainment, occupational status, Townsend deprivation index, alcohol consumption, smoking status, regular exercise, body mass index, and family history of CVD (or diabetes)

In the secondary analysis, we observed that a 1-unit increase in both FP_Frailty and FI_Frailty was significantly associated with an increased risk of CVD (Fig. 3). For instance, in the fully adjusted model, compared with participants having an FP_Frailty score of 0, those having an FP_Frailty score of 1, 2, 3, ≥4 had an increased risk of CVD, with HRs of 1.15 (95% CI: 1.01, 1.30), 1.27 (95% CI: 1.07, 1.50), 2.02 (95% CI: 1.62, 2.53), and 2.53 (95% CI: 1.79, 3.58), respectively. Linear trends were observed for both FP_Frailty and FI_Frailty (all *P* for trend <0.001). Also, we observed that a 1-unit increase in FI_Frailty was associated with a higher risk of incident T2DM (*P* for trend <0.001). We failed to find a similar dose-dependent association between FP_Frailty and incident T2DM (*P* for trend =0.089).

### Sensitivity analyses

**Fig. 4:**
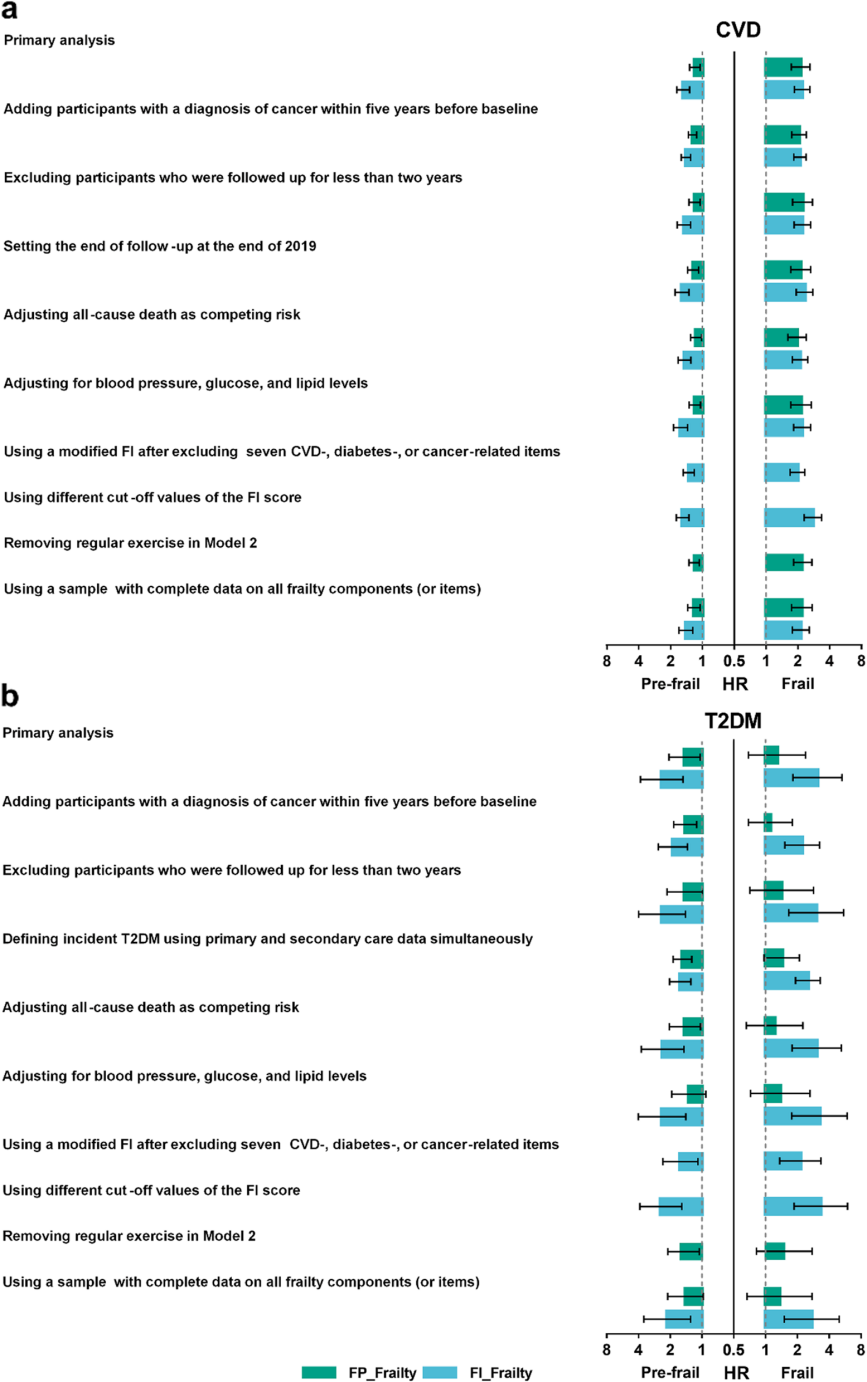
Tornado diagram of sensitivity analyses. CVD, cardiovascular disease; T2DM, type 2 diabetes mellitus; HR, hazard ratio; FP_Frailty, frailty defined by the frailty phenotype; FI_Frailty, frailty defined by the frailty index. **a** Sensitivity analyses of the associations between frailty and incident CVD. **b** Sensitivity analyses of the associations between frailty and incident T2DM. The *x*-axis was log_2_ scaled. The HRs were adjusted for age, sex, ethnicity, educational attainment, occupational status, Townsend deprivation index, alcohol consumption, smoking status, regular exercise, body mass index, and family history of CVD (or diabetes)

Figure 4 shows the robust results of sensitivity analyses. First, we observed that the results did not change substantially when (1) adding participants with a diagnosis of cancer within 5 years before baseline (Additional file 3: Table S2); (2) excluding those who followed up for less than 2 years (Additional file 3: Table S3); (3) setting the end of follow-up at the end of 2019 (Additional file 3: Table S4); (4) defining incident T2DM using primary and secondary care data simultaneously (Additional file 3: Table S5); (5) adjusting for all-cause death as competing risk (Additional file 3: Table S6); and (6) further adjusting for blood pressure, glucose, and lipids levels (Additional file 3: Table S7). Second, when using a modified FI after excluding seven CVD-, diabetes-, or cancer-related items, we discovered similar significant associations between FI_Frailty and incident CVD and T2DM (Additional file 3: Table S8). Also, using different cut-off values of the FI score did not change the association materially (Additional file 3: Table S9). Third, after removing regular exercise in model 2, the associations between FP_Frailty and incident CVD and T2DM remained unchanged (Additional file 3: Table S10). Fourth, when repeating the primary analysis using a sample with complete data on all frailty components (or items), we found that the associations between frailty and risk of CVD were maintained (Additional file 3: Table S11). Finally, we found that most of the individual FP_Frailty components, including slow gait speed, exhaustion, weakness, and unintentional weight loss, were associated with incident CVD, while only weakness was significantly associated with incident T2DM (Additional file 1: Fig. S2).

## Discussion

In this prospective study of long-term cancer survivors, we demonstrated that frailty was significantly associated with an increased risk of developing CVD and T2DM, although discrepancies existed between frailty indicators. With the number of long-term cancer survivors on the rise, our findings suggest that frailty assessment may help to identify the most vulnerable subgroup at a high risk of subsequent CVD and T2DM, and then deliver appropriate disease management, such as prevention and interventive strategies for frailty.

Emerging evidence suggests that cancer survivors experience accelerated aging, and thus the cumulative effect of aging and cancer-related issues may result in a poor prognosis, including adverse health outcomes and low quality of life [15, 41]. In this study, the estimated prevalence of frailty by two frailty indicators was 5.0–15.7%, which is relatively higher than that in the general population from the UKB (FP_Frailty: 3%) [33], but is comparable to that among other cancer survivors (range: 6%-86%) [25]. The high prevalence of frailty among cancer survivors cannot be merely attributed to their older age relative to the general population (mean age=60.4 years vs. 56.9 years). It may rather reveal a faster functional aging process in cancer survivors [42]. This may be partially explained by cancer-related treatments (e.g., the impact of cytotoxic and genotoxic treatments on normal cells) [43, 44] or the shared physiological mechanisms between cancer and aging (e.g., DNA integrity and stability, epigenetic modifications) [45, 46]. As referred to above, accelerated functional aging in cancer survivors, indicated by two frailty indicators (FP_Frailty and FI_Frailty) in this study, was found to be associated with a higher risk of CVD and T2DM. The biological mechanisms underpinning these associations are complicated and remain unexplored. Since frailty involves the dysregulation of multiple systems, it is likely that cancer or cancer-related treatments drive degeneration and decline in body and multiple functions (e.g., grip strength), and in turn predispose patients to adverse chronic diseases. From the aspect of health implications, our study supports the implementation of frailty assessment to identify long-term cancer survivors that are at higher risk of subsequent CVD and T2DM more than 7 years before their occurrence. This is different from frailty screening due to treatment-related toxicity, which most often occurs before cancer treatment in oncological practice. Conversely, building on the findings of this study, cancer patients may largely benefit from being routinely monitored after treatment for the improved identification, prevention, and intervention of frailty.

The positive association of FI_Frailty with incident T2DM was observed, and a series of sensitivity analyses confirmed the robustness of the findings. Nevertheless, we found a null association for FP_Frailty, which is interesting. Firstly, our findings support the differences in predicting outcomes between FP_Frailty and FI_Frailty as demonstrated in previous studies [47–49]. For instance, compared with FP_Frailty, FI_Frailty based on the accumulation of deficits has a relatively stronger association with mortality [47]. Moreover, FI_Frailty was found to identify vulnerable individuals even in a robust subgroup that was categorized by FP_Frailty [50]. These discrepancies may be partially explained by the broader spectrum of health-deficit accumulation measured by FI_Frailty which represents a closer risk profile to the clinical condition [28]. In the clinical setting, the choice of frailty measurements should balance the implementation feasibility and prediction performance. Second, to ensure the precision of incident T2DM, we used primary care data covering about half of the UKB sample, rather than linked hospital data, to identify these incident cases [30]. The null association may be induced by the low statistical power due to the limited sample size determined at the practice level. Third, because FP_Frailty we constructed in this study was adapted from the original definition [18], thus, there may be misclassification. Future research is required to help understand the associations between different frailty indicators and the risk of incident T2DM in long-term cancer survivors.

According to the findings of this study, cancer patients at the pre-frail stage may be a potential subgroup to be targeted. First, over 40% of cancer survivors were identified as pre-frail in our study. Second, not only pre-frailty but also the very early stage of frailty (e.g., a FI score between 0.1 and 0.2) were positively associated with incident CVD and T2DM. Third, as an intermediate transition stage from robust to frailty, the pre-frail stage has the potential to be intervened [18]. For instance, physical exercise training and nutrition supplementation were found to prevent the progression from pre-frailty to frailty [51, 52]. Moreover, these strategies have shown beneficial effects on physical performance and health-related quality of life in pre-frail adults [52, 53]. Hence, taking advantage of the potential intervention opportunity to successfully delay or reverse frailty may have a significant impact on the long-term prognosis of cancer survivors at the early stage of frailty. Nevertheless, evidence on the cost-effectiveness of interventional programs targeting pre-frailty is scarce. On the other hand, our findings still need to be validated. Thus, more efforts are needed to balance resource allocation with health benefits.

The significant strengths of our study included the large sample size of cancer survivors with long follow-up. Furthermore, we applied two widely used frailty indicators; FP_Fraity emphasized frailty as a physical function, while FI_Frailty focused on the accumulation of health defects for the whole organism. In addition, a series of sensitivity analyses were performed to confirm the validity of the findings.

Some limitations in this study should also be noted. First, the information on the stage, grade, and treatment of cancers was unavailable, restricting the exploration of their potential influence on the results. Second, the dataset included limited numbers of cancers other than breast cancer or colorectal cancer; thereby, we were unable to evaluate the effect of cancer type on the findings. Third, we used primary care data to define incident T2DM; however, more than half of cancer survivors did not have primary care data, which may result in low statistical power. Our findings require confirmation when the primary care data was expanded to all the UKB participants in the future. Fourth, the FP_Frailty components we used were adapted from the original definitions. For instance, slow gait speed was assessed by self-reported usual walking pace, which was less reliable than objectively measured gait speed; weight loss was not specified as unintentional. Finally, the majority of UKB participants were White and lived at a higher socioeconomic level [54], and thus, our sample was less representative of all long-term cancer survivors in the UK. A recent study has shown that cancer prevalence in UK Biobank was almost similar to that in the Secure Anonymised Information Linkage (SAIL) databank (an unselected population from Wales) (6.22% vs 5.78%), as was the hazard ratio for all-cause mortality (1.99 vs 1.86) [55]. Also, the frailty prevalence of our sample was comparable to that in the literature [25]. These observations suggest a reduced magnitude of sample selection bias to some extent. However, we caution that our estimates of associations may be subject to collider bias to a lesser extent [56]. Future research on other populations is warranted to validate our results.

## Conclusions

Among long-term cancer survivors, we established that frailty—even at the very early stage, was positively associated with the incidence risk of CVD and T2DM, although there were discrepancies between frailty indicators. While further research is required to validate our findings, they suggest that routine monitoring, prevention, and interventive programs of frailty among cancer survivors may help to prevent late comorbidities and, eventually, improve quality of life. Especially, interventions are recommended to target those at an early stage of frailty when healthcare resources are limited.

## Supplementary Information

**Additional file 1: Fig. S1.** Flow chart of the analytic sample. **Fig. S2.** The associations of individual FP_Frailty components with incident CVD and T2DM among long-term cancer survivors.

**Additional file 2.** Supplementary Methods: FP_Frailty [18, 33].

**Additional file 3: Table S1.** Items of the frailty index from the baseline UK Biobank assessment. **Table S2.** The associations of frailty (three-categorical) with incident CVD and T2DM among total cancer survivors. **Table S3.** The associations of frailty (three-categorical) with incident CVD and T2DM among long-term cancer survivors (followed up for over two years). **Table S4.** The associations of frailty (three-categorical) with incident CVD among long-term cancer survivors when setting the end of follow-up at the end of 2019. **Table S5.** Primary analysis showing the associations of frailty (three-categorical) with incident T2DM (defined using primary care data and secondary care data simultaneously) among long-term cancer survivors (*N*=13,506). **Table S6.** Primary analysis showing the associations of frailty (three-categorical) with incident CVD and T2DM among long-term cancer survivors adjusting for all-cause death as competing risk. **Table S7.** Primary analysis showing the associations of frailty (three-categorical) with incident CVD and T2DM among long-term cancer survivors further adjusting for blood pressure, glucose, and lipids levels. **Table S8.** Primary analysis showing the association of the modified FI_Frailty (42 items) with incident CVD and T2DM among long-term cancer survivors. **Table S9.** Primary analysis showing the association of FI_Frailty (three-categorical, use 0.25 as cut-off value of frailty) with incident CVD and T2DM among long-term cancer survivors. **Table S10.** Primary analysis showing the association of FP_Frailty (three-categorical) with incident CVD and T2DM among long-term cancer survivors in the model without adjustment for regular exercise. **Table S11.** Primary analysis showing the associations of frailty (three-categorical) with incident CVD and T2DM among long-term cancer survivors using a sample with complete data on all frailty components (or items).

## Data Availability

The datasets supporting the conclusions of this article are available in www.ukbiobank.ac.uk/register-apply.

## Abbreviations

BMI: Body mass index
CI: Confidence interval
CVD: Cardiovascular disease
FI: Frailty index
FI_Frailty: Frailty defined by the frailty index
FP_Frailty: Frailty defined by the frailty phenotype
HR: Hazard ratio
ICD-9: The International Statistical Classification of Diseases and Related Health Problems 9th
ICD-10: The International Statistical Classification of Diseases and Related Health Problems 10th
IQRs: Inter-quartile ranges
TDI: Townsend deprivation index
T2DM: Type 2 diabetes mellitus
UKB: UK Biobank
