# Supplementary material for: Association of frailty with the incidence risk of cardiovascular disease and type 2 diabetes mellitus in long-term cancer survivors: a prospective cohort study
Source: BMC Med. 2023 Feb 24;21:74. doi: 10.1186/s12916-023-02774-1 (PMC9951842; doi:10.1186/s12916-023-02774-1)
Supplement: Supplementary file 1 — Additional file 1: Fig. S1. Flow chart of the analytic sample. Fig. S2. The associations of individual FP_Frailty components with incident CVD and T2DM among long-term cancer survivors. [file 12916_2023_2774_MOESM1_ESM.docx]

**Additional file 1**


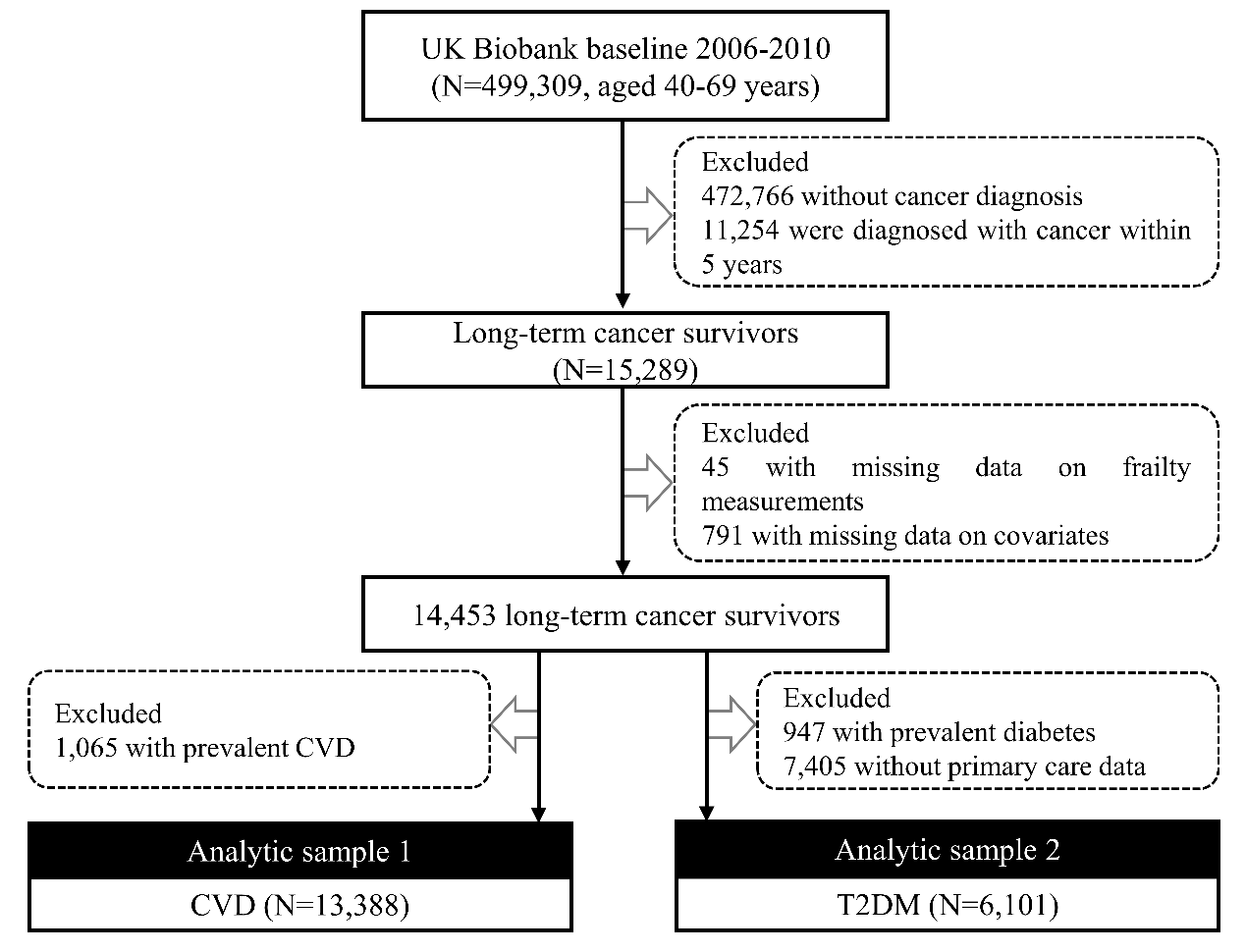


**Fig. S1. Flow chart of the analytic sample.** CVD, cardiovascular disease; T2DM, type 2 diabetes mellitus.


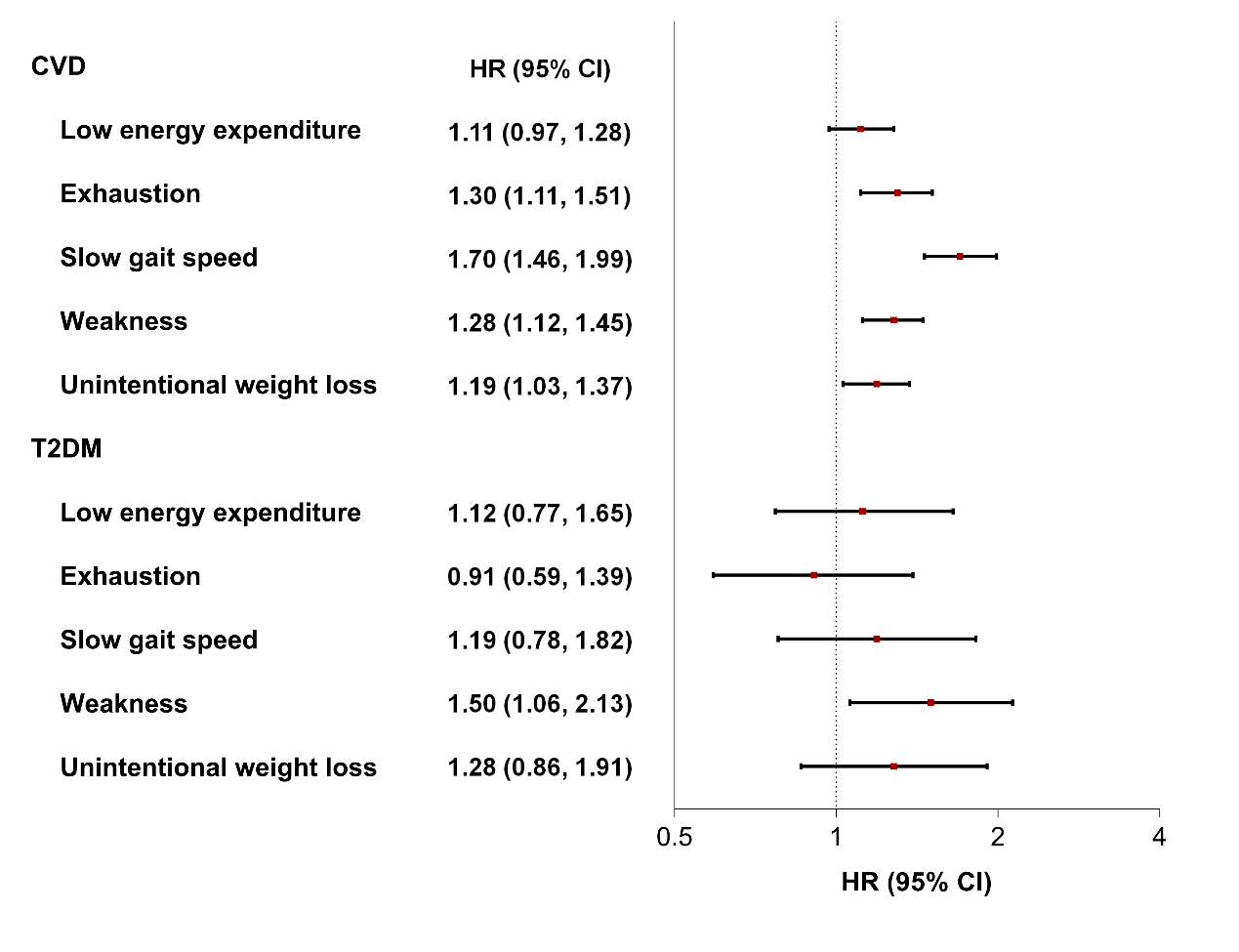
 **Fig. S2. The associations of individual FP_Frailty components with incident CVD and T2DM among long-term cancer survivors.** The x-axis was log_2_ scaled. The HRs were adjusted for age, sex, ethnicity, educational attainment, occupational status, Townsend deprivation index, alcohol consumption, smoking status, regular exercise, body mass index, and family history of CVD (or diabetes). The prevalence rates of low energy expenditure, exhaustion, slow gait speed, weakness, and unintentional weight loss were 25.5%, 12.4%, 9.3%, 17.3%, and 15.3% respectively, in the analytic sample for incident CVD. The prevalence rates of low energy expenditure, exhaustion, slow gait speed, weakness, and unintentional weight loss were 25.3%, 12.4%, 9.7%, 16.8%, and 14.3% respectively, in the analytic sample for incident T2DM. CVD, cardiovascular disease; T2DM, type 2 diabetes mellitus; HR, hazard ratio; CI, confidence interval; FP_Frailty, Frailty defined by the frailty phenotype.
