# Supplementary material for: Association of frailty with the incidence risk of cardiovascular disease and type 2 diabetes mellitus in long-term cancer survivors: a prospective cohort study
Source: BMC Med. 2023 Feb 24;21:74. doi: 10.1186/s12916-023-02774-1 (PMC9951842; doi:10.1186/s12916-023-02774-1)
Supplement: Supplementary file 2 — Additional file 2. Supplementary Methods: FP_Frailty [18, 33]. [file 12916_2023_2774_MOESM2_ESM.docx]

**Additional file 2**

**Supplementary Methods: FP_Frailty**

The frailty phenotype proposed by Fried (FP_Frailty) [1], was assessed using five components, including low energy expenditure, exhaustion, slow gait speed, weakness, and unintentional weight loss [2].

1. Low energy expenditure: Participants were asked “In the last 4 weeks, did you spend any time doing the light DIY activity, heavy DIY activity, or strenuous sports?” Participants answering “None or light activity with a frequency of once per week or less” were categorized as frail by the low energy expenditure criteria.
2. Exhaustion: Participants were asked “Over the past two weeks, how often have you felt tired or had little energy?” Participants answering “more than half the days or nearly every day” were categorized as frail by the exhaustion criteria.
3. Slow gait speed: Participants were asked “How would you describe your usual walking pace?” Participants answering “Slow pace” were categorized as frail by the slow gait speed criteria.
4. Weakness: Weakness was measured by grip strength using a Jamar J00105 hydraulic hand dynamometer (Lafayette Instrument, Lafayette, IN, USA). Participants were asked to complete a grip assessment for both hands once. The maximal value of the right and left hands was used. Then, sex and body mass index (BMI) adjusted cutoffs were used to define weakness as following: ① Male: ≤29 kg for BMI ≤24 kg/m^2^; ≤30 kg for BMI 24.1-26 kg/m^2^; ≤30 kg for BMI 26.1-28 kg/m^2^; or ≤32 kg for BMI >28 kg/m^2^; ② Female: ≤17 kg for BMI ≤23 kg/m^2^; ≤17.3 kg for BMI 23.1-26 kg/m^2^; ≤18 kg for BMI 26.1-29 kg/m^2^; or ≤21 kg for BMI >29 kg/m^2^.
5. Unintentional weight loss: Participants were asked: “Compared with one year ago, has your weight changed?” Participants answering “Yes, loss weight” were categorized as frail by the unintentional weight loss criteria.

**References:**

1. Fried LP, Tangen CM, Walston J, Newman AB, Hirsch C, Gottdiener J, et al. Frailty in older adults: evidence for a phenotype. J Gerontol A Biol Sci Med Sci. 2001;56(3):M146-156.

2. Hanlon P, Nicholl BI, Jani BD, Lee D, McQueenie R, Mair FS. Frailty and pre-frailty in middle-aged and older adults and its association with multimorbidity and mortality: a prospective analysis of 493 737 UK Biobank participants. Lancet Public Health. 2018;3(7):e323-e332.
