# Supplementary material for: Association of frailty with the incidence risk of cardiovascular disease and type 2 diabetes mellitus in long-term cancer survivors: a prospective cohort study
Source: BMC Med. 2023 Feb 24;21:74. doi: 10.1186/s12916-023-02774-1 (PMC9951842; doi:10.1186/s12916-023-02774-1)
Supplement: Supplementary file 3 — Additional file 3: Table S1. Items of the frailty index from the baseline UK Biobank assessment. Table S2. The associations of frailty (three-categorical) with incident CVD and T2DM among total cancer survivors. Table S3. The associations of frailty (three-categorical) with incident CVD and T2DM among long-term cancer survivors (followed up for over two years). Table S4. The associations of frailty (three-categorical) with incident CVD among long-term cancer survivors when setting the end of follow-up at the end of 2019. Table S5. Primary analysis showing the associations of frailty (three-categorical) with incident T2DM (defined using primary care data and secondary care data simultaneously) among long-term cancer survivors (N=13,506). Table S6. Primary analysis showing the associations of frailty (three-categorical) with incident CVD and T2DM among long-term cancer survivors adjusting for all-cause death as competing risk. Table S7. Primary analysis showing the associations of frailty (three-categorical) with incident CVD and T2DM among long-term cancer survivors further adjusting for blood pressure, glucose, and lipids levels. Table S8. Primary analysis showing the association of the modified FI_Frailty (42 items) with incident CVD and T2DM among long-term cancer survivors. Table S9. Primary analysis showing the association of FI_Frailty (three-categorical, use 0.25 as cut-off value of frailty) with incident CVD and T2DM among long-term cancer survivors. Table S10. Primary analysis showing the association of FP_Frailty (three-categorical) with incident CVD and T2DM among long-term cancer survivors in the model without adjustment for regular exercise. Table S11. Primary analysis showing the associations of frailty (three-categorical) with incident CVD and T2DM among long-term cancer survivors using a sample with complete data on all frailty components (or items). [file 12916_2023_2774_MOESM3_ESM.docx]

**Additional file 3**

**Table S1. Items of the frailty index from the baseline UK Biobank assessment.**

| **Type of deficit** | **No.** | **Item description** | **Categories** |
| --- | --- | --- | --- |
| *Sensory* | 1 | Glaucoma | 0: no; 1: yes |
|  | 2 | Cataracts | 0: no; 1: yes |
|  | 3 | Hearing difficulty | 0: no; 1: yes, completely deaf |
|  |  |  |  |
| *Cranial* | 4 | Migraine | 0: no; 1: yes |
|  | 5 | Dental problems | 0: none; 1: any of ulcers, painful gums, bleeding gums, loose teeth, toothache, dentures |
| *Mental wellbeing* | 6 | Self-rated health | 0: excellent; 0.25: good; 0.5: fair; 1: poor |
|  | 7 | Fatigue: frequency of tiredness/lethargy in the last two weeks | 0: not at all; 0.25: several days; 0.5: more than half; 1: nearly every day |
|  | 8 | Sleep: experience of sleeplessness/insomnia | 0: never/rarely; 0.5: sometimes; 1: usually |
|  | 9 | Depressed feelings: frequency in last two weeks | 0: not at all; 0.5: several days; 0.75: more than half, nearly every day |
|  |  |  |  |
|  | 10 | Self-described nervous personality | 0: no; 1: yes |
|  | 11 | Severe anxiety/panic attacks | 0: no; 1: yes |
|  | 12 | Common to feel loneliness | 0: no; 1: yes |
|  | 13 | Sense of misery (ever/never) | 0: no; 1: yes |
| *Infirmity* | 14 | Infirmity: long-standing illness or disability | 0: no; 1: yes |
|  | 15 | Falls in last year | 0: no falls; 0.5: one fall; 1: more than one |
|  | 16 | Fractures/broken bones in the last five years | 0: no; 1: yes |
| *Cardiometabolic* | 17 | Diabetes | 0: no; 1: yes |
|  | 18 | Myocardial infarction | 0: no; 1: yes |
|  | 19 | Angina | 0: no; 1: yes |
|  | 20 | Stroke | 0: no; 1: yes |
|  | 21 | High blood pressure | 0: no; 1: yes |
|  | 22 | Hypothyroidism | 0: no; 1: yes |
|  | 23 | Deep-vein thrombosis | 0: no; 1: yes |
|  | 24 | High cholesterol | 0: no; 1: yes |
| *Respiratory* | 25 | Breathing: wheeze in last year | 0: no; 1: yes |
|  | 26 | Pneumonia | 0: no; 1: yes |
|  | 27 | Chronic bronchitis/emphysema | 0: no; 1: yes |
|  | 28 | Asthma | 0: no; 1: yes |
| *Musculoskeletal* | 29 | Rheumatoid arthritis | 0: no; 1: yes |
|  | 30 | Osteoarthritis | 0: no; 1: yes |
|  | 31 | Gout | 0: no; 1: yes |
|  | 32 | Osteoporosis | 0: no; 1: yes |
| *Immunological* | 33 | Hayfever, allergic rhinitis, or eczema | 0: no; 1: yes |
|  | 34 | Psoriasis | 0: no; 1: yes |
| *Cancer* | 35 | Any cancer diagnosis | 0: no; 1: yes |
|  | 36 | Multiple cancers diagnosed (number reported) | 0: no cancer or single cancer; 1: multiple cancers |
| *Pain* | 37 | Chest pain | 0: no; 1: yes |
|  | 38 | Head and/or neck pain | 0: no; 1: yes (combining responses to pain in head and neck/shoulders) |
|  | 39 | Back pain | 0: no; 1: yes |
|  | 40 | Stomach/abdominal pain | 0: no; 1: yes |
|  | 41 | Hip pain | 0: no; 1: yes |
|  | 42 | Knee pain | 0: no; 1: yes |
|  | 43 | Whole-body pain | 0: no; 1: yes |
|  | 44 | Facial pain | 0: no; 1: yes |
|  | 45 | Sciatica | 0: no; 1: yes |
| *Gastrointestinal* | 46 | Gastric reflux | 0: no; 1: yes |
|  | 47 | Hiatus hernia | 0: no; 1: yes |
|  | 48 | Gall stones | 0: no; 1: yes |
|  | 49 | Diverticulitis | 0: no; 1: yes |

**Table S2. The associations of frailty (three-categorical) with incident CVD and T2DM among total cancer survivors.**

|  | **No. of events/No. of participants** | **Model 1** | **Model 2** |
| --- | --- | --- | --- |
|  |  | **HR (95% CI)** | **HR (95% CI)** |
| **CVD (N=23,071)** | | | |
| **FP_Frailty** |  |  |  |
| Non-frail | 956/11,072 | Ref. | Ref. |
| Pre-frail | 1,141/10,719 | 1.37 (1.25, 1.49) | 1.24 (1.13, 1.35) |
| Frail | 237/1,280 | 2.80 (2.43, 3.23) | 2.05 (1.75, 2.40) |
| P for trend | — | <0.001 | <0.001 |
| **FI_Frailty** |  |  |  |
| Non-frail | 507/7,345 | Ref. | Ref. |
| Pre-frail | 1,272/12,264 | 1.58 (1.42, 1.75) | 1.43 (1.29, 1.58) |
| Frail | 555/3,462 | 2.67 (2.37, 3.01) | 2.09 (1.83, 2.38) |
| P for trend | — | <0.001 | <0.001 |
| **T2DM (N=10,520)** | | | |
| **FP_Frailty** |  |  |  |
| Non-frail | 111/5,103 | Ref. | Ref. |
| Pre-frail | 203/4,849 | 2.05 (1.63, 2.59) | 1.44 (1.12, 1.85) |
| Frail | 29/568 | 2.67 (1.78, 4.02) | 1.11 (0.69, 1.79) |
| P for trend | — | <0.001 | 0.072 |
| **FI_Frailty** |  |  |  |
| Non-frail | 49/3,250 | Ref. | Ref. |
| Pre-frail | 200/5,616 | 2.37 (1.73, 3.23) | 1.88 (1.37, 2.59) |
| Frail | 94/1,654 | 3.93 (2.78, 5.55) | 2.22 (1.52, 3.24) |
| P for trend | — | <0.001 | <0.001 |

Linear trends were tested using FP_Frailty and FI_Frailty (three-categorical) as continuous variables.

CVD, cardiovascular disease; T2DM, type 2 diabetes mellitus; HR, hazard ratio; CI, confidence interval; FP_Frailty, Frailty defined by the frailty phenotype; FI_Frailty, Frailty defined by the frailty index.

Model 1 was adjusted for age, and sex.

Model 2 was further adjusted for ethnicity, educational attainment, occupational status, Townsend deprivation index, alcohol consumption, smoking status, regular exercise, body mass index, and family history of CVD (or diabetes).

**Table S3. The associations of frailty (three-categorical) with incident CVD and T2DM among long-term cancer survivors (followed up for over two years).**

|  | **No. of events/No. of participants** | **Model 1** | **Model 2** |
| --- | --- | --- | --- |
|  |  | **HR (95% CI)** | **HR (95% CI)** |
| **CVD (N=13,046)** | | | |
| **FP_Frailty** |  |  |  |
| Non-frail | 513/6,473 | Ref. | Ref. |
| Pre-frail | 573/5,937 | 1.31 (1.16, 1.47) | 1.18 (1.05, 1.34) |
| Frail | 127/636 | 3.04 (2.50, 3.69) | 2.21 (1.78, 2.74) |
| P for trend | — | <0.001 | <0.001 |
| **FI_Frailty** |  |  |  |
| Non-frail | 252/4,193 | Ref. | Ref. |
| Pre-frail | 658/6,874 | 1.65 (1.43, 1.91) | 1.49 (1.29, 1.73) |
| Frail | 303/1,979 | 2.82 (2.39, 3.34) | 2.20 (1.84, 2.63) |
| P for trend | — | <0.001 | <0.001 |
| **T2DM (N=5,984)** | | | |
| **FP_Frailty** |  |  |  |
| Non-frail | 50/2,998 | Ref. | Ref. |
| Pre-frail | 83/2,697 | 1.96 (1.38, 2.78) | 1.46 (0.99, 2.14) |
| Frail | 14/289 | 3.07 (1.70, 5.54) | 1.42 (0.71, 2.83) |
| P for trend | — | <0.001 | 0.090 |
| **FI_Frailty** |  |  |  |
| Non-frail | 18/1,879 | Ref. | Ref. |
| Pre-frail | 86/3,179 | 2.81 (1.69, 4.68) | 2.38 (1.43, 3.97) |
| Frail | 43/926 | 4.92 (2.83, 8.56) | 3.01 (1.66, 5.47) |
| P for trend | — | <0.001 | <0.001 |

Linear trends were tested using FP_Frailty and FI_Frailty (three-categorical) as continuous variables.

CVD, cardiovascular disease; T2DM, type 2 diabetes mellitus; HR, hazard ratio; CI, confidence interval; FP_Frailty, Frailty defined by the frailty phenotype; FI_Frailty, Frailty defined by the frailty index.

Model 1 was adjusted for age, and sex.

Model 2 was further adjusted for ethnicity, educational attainment, occupational status, Townsend deprivation index, alcohol consumption, smoking status, regular exercise, body mass index, and family history of CVD (or diabetes).

**Table S4. The associations of frailty (three-categorical) with incident CVD among long-term cancer survivors when setting the end of follow-up at the end of 2019.**

|  | **No. of events/No. of participants** | **Model 1** | **Model 2** |
| --- | --- | --- | --- |
|  |  | **HR (95% CI)** | **HR (95% CI)** |
| **FP_Frailty** |  |  |  |
| Non-frail | 504/6,598 | Ref. | Ref. |
| Pre-frail | 585/6,114 | 1.34 (1.19, 1.51) | 1.22 (1.08, 1.38) |
| Frail | 125/676 | 2.90 (2.39, 3.54) | 2.12 (1.71, 2.64) |
| P for trend | — | <0.001 | <0.001 |
| **FI_Frailty** |  |  |  |
| Non-frail | 241/4,247 | Ref. | Ref. |
| Pre-frail | 664/7,077 | 1.72 (1.48, 1.99) | 1.56 (1.34, 1.81) |
| Frail | 309/2,064 | 2.92 (2.47, 3.46) | 2.31 (1.93, 2.76) |
| P for trend | — | <0.001 | <0.001 |

Linear trends were tested using FP_Frailty and FI_Frailty (three-categorical) as continuous variables.

CVD, cardiovascular disease; HR, hazard ratio; CI, confidence interval; FP_Frailty, Frailty defined by the frailty phenotype; FI_Frailty, Frailty defined by the frailty index.

Model 1 was adjusted for age, and sex.

Model 2 was further adjusted for ethnicity, educational attainment, occupational status, Townsend deprivation index, alcohol consumption, smoking status, regular exercise, body mass index, and family history of CVD (or diabetes).

**Table S5. Primary analysis showing the associations of frailty (three-categorical) with incident T2DM (defined using primary care data and secondary care data simultaneously) among long-term cancer survivors (N=13,506).**

|  | **No. of events/No. of participants** | **Model 1** | **Model 2** |
| --- | --- | --- | --- |
|  |  | **HR (95% CI)** | **HR (95% CI)** |
| **FP_Frailty** | | | |
| Non-frail | 187/6,708 | Ref. | Ref. |
| Pre-frail | 339/6,130 | 2.15 (1.80, 2.57) | 1.53 (1.25, 1.87) |
| Frail | 59/668 | 3.84 (2.86, 5.16) | 1.43 (0.97, 2.09) |
| P for trend | — | <0.001 | <0.001 |
| **FI_Frailty** | | | |
| Non-frail | 95/4,242 | Ref. | Ref. |
| Pre-frail | 302/7,118 | 1.97 (1.56, 2.48) | 1.60 (1.27, 2.02) |
| Frail | 188/2,146 | 4.32 (3.37, 5.54) | 2.52 (1.92, 3.29) |
| P for trend | — | <0.001 | <0.001 |

Linear trends were tested using FP_Frailty and FI_Frailty (three-categorical) as continuous variables.

T2DM, type 2 diabetes mellitus; HR, hazard ratio; CI, confidence interval; FP_Frailty, Frailty defined by the frailty phenotype; FI_Frailty, Frailty defined by the frailty index.

Model 1 was adjusted for age, and sex.

Model 2 was further adjusted for ethnicity, educational attainment, occupational status, Townsend deprivation index, alcohol consumption, smoking status, regular exercise, body mass index, and family history of DM.

**Table S6. Primary analysis showing the associations of frailty (three-categorical) with incident CVD and T2DM among long-term cancer survivors adjusting for all-cause death as competing risk.**

|  | **No. of events/No. of participants** | **Model 1** | **Model 2** |
| --- | --- | --- | --- |
|  |  | **HR (95% CI)** | **HR (95% CI)** |
| **CVD** | | | |
| **FP_Frailty** |  |  |  |
| Non-frail | 582/6,598 | Ref. | Ref. |
| Pre-frail | 654/6,114 | 1.27 (1.14, 1.42) | 1.15 (1.02, 1.29) |
| Frail | 144/676 | 2.68 (2.23, 3.22) | 1.95 (1.60, 2.39) |
| P for trend | — | <0.001 | <0.001 |
| **FI_Frailty** |  |  |  |
| Non-frail | 282/4,247 | Ref. | Ref. |
| Pre-frail | 753/7,077 | 1.62 (1.41, 1.86) | 1.47 (1.28, 1.69) |
| Frail | 345/2,064 | 2.67 (2.28, 3.12) | 2.10 (1.77, 2.48) |
| P for trend | — | <0.001 | <0.001 |
| **T2DM** | | | |
| **FP_Frailty** |  |  |  |
| Non-frail | 62/3,039 | Ref. | Ref. |
| Pre-frail | 108/2,755 | 2.03 (1.48, 2.78) | 1.45 (1.03, 2.03) |
| Frail | 17/307 | 2.70 (1.58, 4.62) | 1.22 (0.66, 2.25) |
| P for trend | — | <0.001 | 0.114 |
| **FI_Frailty** |  |  |  |
| Non-frail | 22/1,894 | Ref. | Ref. |
| Pre-frail | 109/3,249 | 2.84 (1.79, 4.51) | 2.36 (1.48, 3.75) |
| Frail | 56/958 | 5.04 (3.06, 8.29) | 3.04 (1.78, 5.18) |
| P for trend | — | <0.001 | <0.001 |

Linear trends were tested using FP_Frailty and FI_Frailty (three-categorical) as continuous variables.

CVD, cardiovascular disease; T2DM, type 2 diabetes mellitus; HR, hazard ratio; CI, confidence interval; FP_Frailty, Frailty defined by the frailty phenotype; FI_Frailty, Frailty defined by the frailty index.

Model 1 was adjusted for age, and sex.

Model 2 was further adjusted for ethnicity, educational attainment, occupational status, Townsend deprivation index, alcohol consumption, smoking status, regular exercise, body mass index, and family history of CVD (or diabetes).

**Table S7. Primary analysis showing the associations of frailty (three-categorical) with incident CVD and T2DM among long-term cancer survivors further adjusting for blood pressure, glucose, and lipids levels.**

|  | **Model 3** |
| --- | --- |
|  | **HR (95% CI)** |
| **CVD** | |
| **FP_Frailty** |  |
| Non-frail | Ref. |
| Pre-frail | 1.18 (1.04, 1.33) |
| Frail | 2.14 (1.71, 2.67) |
| P for trend | <0.001 |
| **FI_Frailty** |  |
| Non-frail | Ref. |
| Pre-frail | 1.61 (1.38, 1.87) |
| Frail | 2.19 (1.82, 2.63) |
| P for trend | <0.001 |
| **T2DM** | |
| **FP_Frailty** |  |
| Non-frail | Ref. |
| Pre-frail | 1.33 (0.92, 1.93) |
| Frail | 1.38 (0.72, 2.63) |
| P for trend | 0.147 |
| **FI_Frailty** |  |
| Non-frail | Ref. |
| Pre-frail | 2.38 (1.42, 4.01) |
| Frail | 3.23 (1.77, 5.88) |
| P for trend | <0.001 |

Linear trends were tested using FP_Frailty and FI_Frailty (three-categorical) as continuous variables.

CVD, cardiovascular disease; T2DM, type 2 diabetes mellitus; HR, hazard ratio; CI, confidence interval; FP_Frailty, Frailty defined by the frailty phenotype; FI_Frailty, Frailty defined by the frailty index.

Model 3 was further adjusted for systolic blood pressure, diastolic blood pressure, glucose, cholesterol, triglycerides, high-density lipoprotein, and low-density lipoprotein based on Model 2.

**Table S8. Primary analysis showing the association of the modified FI_frailty (42 items) with incident CVD and T2DM among long-term cancer survivors.**

|  | **No. of events/No. of participants** | **Model 1** | **Model 2** |
| --- | --- | --- | --- |
|  |  | **HR (95% CI)** | **HR (95% CI)** |
| **CVD** | | | |
| Non-frail | 431/5,623 | Ref. | Ref. |
| Pre-frail | 639/5,909 | 1.50 (1.33, 1.70) | 1.34 (1.19, 1.52) |
| Frail | 310/1,856 | 2.54 (2.20, 2.95) | 1.98 (1.69, 2.32) |
| P for trend | — | <0.001 | <0.001 |
| **T2DM** | | | |
| Non-frail | 39/2,295 | Ref. | Ref. |
| Pre-frail | 94/2,850 | 1.96 (1.35, 2.86) | 1.60 (1.09, 2.34) |
| Frail | 54/956 | 3.51 (2.32, 5.32) | 2.13 (1.36, 3.34) |
| P for trend | — | <0.001 | <0.001 |

Linear trend was tested using FI_Frailty (three-categorical) as a continuous variable.

FI_Frailty, Frailty defined by the frailty index; CVD, cardiovascular disease; T2DM, type 2 diabetes mellitus; HR, hazard ratio; CI, confidence interval.

Model 1 was adjusted for age, and sex.

Model 2 was further adjusted for ethnicity, educational attainment, occupational status, Townsend deprivation index, alcohol consumption, smoking status, regular exercise, body mass index, and family history of CVD (or diabetes).

**Table S9. Primary analysis showing the association of FI_Frailty (three-categorical, use 0.25 as the cut-off value of frailty) with incident CVD and T2DM among long-term cancer survivors.**

|  | **No. of events/No. of participants** | **Model 1** | **Model 2** |
| --- | --- | --- | --- |
|  |  | **HR (95% CI)** | **HR (95% CI)** |
| **CVD** | | | |
| Non-frail | 282/4,247 | Ref. | Ref. |
| Pre-frail | 884/8,094 | 1.73 (1.51, 1.97) | 1.54 (1.34, 1.76) |
| Frail | 214/1,047 | 3.67 (3.07, 4.39) | 2.77 (2.29, 3.35) |
| P for trend | — | <0.001 | <0.001 |
| **T2DM** | | | |
| Non-frail | 22/1,894 | Ref. | Ref. |
| Pre-frail | 131/3,687 | 3.07 (1.95, 4.84) | 2.45 (1.55, 3.88) |
| Frail | 34/520 | 5.80 (3.38, 9.96) | 3.32 (1.86, 5.95) |
| P for trend | — | <0.001 | <0.001 |

Linear trend was tested using FI_Frailty (three-categorical) as a continuous variable.

FI_Frailty, Frailty defined by the frailty index; CVD, cardiovascular disease; T2DM, type 2 diabetes mellitus; HR, hazard ratio; CI, confidence interval.

Model 1 was adjusted for age, and sex.

Model 2 was further adjusted for ethnicity, educational attainment, occupational status, Townsend deprivation index, alcohol consumption, smoking status, regular exercise, body mass index, and family history of CVD (or diabetes).

**Table S10. Primary analysis showing the association of FP_Frailty (three-categorical) with incident CVD and T2DM among long-term cancer survivors in the model without adjustment for regular exercise.**

|  | **No. of events/No. of participants** | **Model 4** |
| --- | --- | --- |
|  |  | **HR (95% CI)** |
| **CVD** | | |
| Non-frail | 582/6,598 | Ref. |
| Pre-frail | 654/6,114 | 1.20 (1.07, 1.34) |
| Frail | 144/676 | 2.21 (1.82, 2.70) |
| P for trend | — | <0.001 |
| **T2DM** | | |
| Non-frail | 62/3,039 | Ref. |
| Pre-frail | 108/2,755 | 1.52 (1.09, 2.13) |
| Frail | 17/307 | 1.50 (0.82, 2.75) |
| P for trend | — | 0.025 |

Linear trend was tested using FP_Frailty (three-categorical) as a continuous variable.

FP_Frailty, Frailty defined by the frailty phenotype; CVD, cardiovascular disease; T2DM, type 2 diabetes mellitus; HR, hazard ratio; CI, confidence interval.

Model 4 was adjusted for age, sex, ethnicity, educational attainment, occupational status, Townsend deprivation index, alcohol consumption, smoking status, body mass index, and family history of CVD (or diabetes).

**Table S11. Primary analysis showing the associations of frailty (three-categorical) with incident CVD and T2DM among long-term cancer survivors using a sample with complete data on all frailty components (or items).**

|  | **No. of events/No. of participants** | **Model 1** | **Model 2** |
| --- | --- | --- | --- |
|  |  | **HR (95% CI)** | **HR (95% CI)** |
| **CVD** | | | |
| **FP_Frailty (N=10,597)** | | | |
| Non-frail | 433/5,040 | Ref. | Ref. |
| Pre-frail | 528/4,975 | 1.34 (1.18, 1.52) | 1.20 (1.05, 1.37) |
| Frail | 124/582 | 3.06 (2.50, 3.73) | 2.17 (1.74, 2.73) |
| P for trend | — | <0.001 | <0.001 |
| **FI_Frailty (N=11,011)** | | | |
| Non-frail | 251/3,644 | Ref. | Ref. |
| Pre-frail | 595/5,741 | 1.57 (1.35, 1.81) | 1.43 (1.23, 1.66) |
| Frail | 270/1,626 | 2.71 (2.28, 3.22) | 2.13 (1.77, 2.56) |
| P for trend | — | <0.001 | <0.001 |
| **T2DM** | | | |
| **FP_Frailty (N=4800)** | | | |
| Non-frail | 19/1,616 | Ref. | Ref. |
| Pre-frail | 82/2,659 | 2.02 (1.39, 2.94) | 1.43 (0.97, 2.11) |
| Frail | 38/733 | 3.51 (1.96, 6.29) | 1.35 (0.67, 2.74) |
| P for trend | — | <0.001 | 0.148 |
| **FI_Frailty (N=5008)** | | | |
| Non-frail | 19/1,616 | Ref. | Ref. |
| Pre-frail | 82/2,659 | 2.64 (1.60, 4.36) | 2.13 (1.28, 3.54) |
| Frail | 38/733 | 4.59 (2.63, 8.01) | 2.73 (1.50, 4.97) |
| P for trend | — | <0.001 | <0.001 |

Linear trends were tested using FP_Frailty and FI_Frailty (three-categorical) as continuous variables.

CVD, cardiovascular disease; T2DM, type 2 diabetes mellitus; HR, hazard ratio; CI, confidence interval; FP_Frailty, Frailty defined by the frailty phenotype; FI_Frailty, Frailty defined by the frailty index.

Model 1 was adjusted for age, and sex.

Model 2 was further adjusted for ethnicity, educational attainment, occupational status, Townsend deprivation index, alcohol consumption, smoking status, regular exercise, body mass index, and family history of CVD (or diabetes).
